# Association between the rs12255372 variant of the *TCF7L2* gene and obesity in a Cameroonian population

**DOI:** 10.1186/s13104-015-1661-3

**Published:** 2015-11-25

**Authors:** Elvis Ndonwi Ngwa, Eugene Sobngwi, Barbara Atogho-Tiedeu, Jean Jacques N. Noubiap, Olivier Sontsa Donfack, Magellan Guewo-Fokeng, Edith Pascale Mato Mofo, Priscille Pokam Fosso, Eric Djahmeni, Rosine Djokam-Dadjeu, Marie-Solange Evehe, Folefac Aminkeng, Wilfred F. Mbacham, Jean Claude Mbanya

**Affiliations:** Department of Biochemistry, Faculty of Science, University of Yaoundé I, Yaoundé, Cameroon; Laboratory for Molecular Medicine and Metabolism, Biotechnology Center, University of Yaoundé I, Yaoundé, Cameroon; Department of Internal Medicine and Specialties, Faculty of Medicine and Biomedical Sciences, University of Yaoundé I, Yaoundé, Cameroon; National Obesity Center, Yaoundé Central Hospital, Yaoundé, Cameroon; Department of Medicine, Groote Schuur Hospital and University of Cape Town, Cape Town, South Africa; Medical Diagnostic Center, Yaoundé, Cameroon; The Canadian Pharmacogenomics Network for Drug Safety (CPNDS), Center for Molecular Medicine and Therapeutics, Department of Medical Genetics, University of British Columbia, Vancouver, Canada; Laboratory for Public Health Research Biotechnologies, Biotechnology Center, University of Yaoundé I, Yaoundé, Cameroon

**Keywords:** Obesity, Transcription factor 7-like 2 (*TCF7L2*), Cameroon, Sub-Saharan Africa

## Abstract

**Background:**

The transcription factor 7-like 2 (*TCF7L2*) is one of the genes that have been identified as possible determinants of diabetes which is associated with obesity. Data on the genetic causes of obesity in sub-Saharan African populations are very scares. The aim of this study was to assess the association between the transcription factor 7-like 2 (*TCF7L2*) gene polymorphism (rs12255372 G/T) and obesity and weight-related traits in a Cameroonian population.

**Methods:**

A case–control study was conducted on 35 obese and 30 non-obese Cameroonian adults. *TCF7L2* rs12255372 genotypes were determined using PCR–RFLP and correlated with BMI and weight-related traits.

**Results:**

No significant association was observed between the rs12255372 T allele (χ^2^ = 0.0684, p = 0.79) or the TT genotype (χ^2^ = 0.372, p = 0.54) of the TCF7L2 gene and obesity in the Cameroonian population. However, amongst the weight-related traits, triglycerides were significantly associated with the T risk allele of the TCF7L2 gene (p = 0.012).

**Conclusion:**

This study on Cameroonian subjects replicates the absence of association between the *TCF7L2* rs12255372 variant and obesity as observed in European and American populations.

## Background

The rising prevalence of overweight and obesity constitutes a global pandemic. The number of overweight and obese individuals increased from 857 million in 1980, to 2.1 billion in 2013 [1]. In 2010, overweight and obesity were estimated to cause 3.4 million deaths, 4 % of years of life lost, and 4 % of disability-adjusted life-years (DALYs) worldwide [2]. In Cameroon, the prevalence of overweight or obesity among adults aged ≥15 years increased from 22.5 % in 2002 to 26 % in 2006 [3, 4].

Obesity results from an imbalance between energy intake and expenditure which is influenced by a variety of multiple factors that could be environmental, psychosocial and genetic [5]. Although this epidemic is attributable to the trend of decreased physical activity and increased calorie intake, these external factors play out on a genetic background that determines body mass and susceptibility to obesity-related diseases [6] and so, common forms of obesity have a strong hereditary component. Depending on the population examined, these genetic factors may account for 6–85 % of the cases [7]. However, the genetic pathways that contribute to obesity have not yet been elucidated.

The transcription factor 7-like 2 (*TCF7L2*) gene is one of those that have been identified as possible determinants of type 2 diabetes mellitus (T2DM). It codes for a transcription factor with a high-mobility box which is one of the components of the Wingless type (Wnt) signaling pathway, initially characterized in colon cancer and in the embryonic development of some organisms such as Drosophila [8]. Wnt pathways are a group of signal transduction pathways made of proteins that pass signals from outside of a cell through cell surface receptors to the inside of the cell. Three have been characterized including the canonical Wnt pathway, the non-canonical planar cell polarity pathway and the non-canonical Wnt/calcium pathway. It has been shown that in adipocytes, the canonical Wnt-signaling via *TCF7L2* down-regulates adipogenesis [9]. Although *TCF7L2* was not identified as a risk factor for obesity in a study involving European populations, its effect on the risk for T2DM was modulated by obesity [10]. Additionally, a study revealed that the rs12255372 variant of *TCF7L2* was protective for obesity in Mexican children [11]. These findings warrant further investigation of the role of *TCF7L2* in obesity among various ethnic populations. We previously reported on the rs12255372 (G/T) and rs7903146 (C/T) polymorphisms of *TCF7L2* as risk factors for type 2 diabetes mellitus in a Cameroonian population [12, 13], however to the best of our knowledge, the association between rs12255372 (G/T) and obesity has not been explored in any African population till date. The aim of this study therefore was to assess the possible association of the *TCF7L2* rs12255372 polymorphism with obesity and weight-related traits in a Cameroonian population. Findings may contribute to further investigations for the understanding of the causal role of heredity in obesity which may foster successful interventions and treatments.

## Methods

### Study population

A case–control study was conducted that involved 35 obese (BMI ≥ 30 kg/m^2^) and 30 non-obese (18 ≤ BMI < 25 kg/m^2^) adults of Cameroonian origin, aged 20 years and above. Obese patients were recruited from the Outpatients Clinic of the National Obesity Center of the Yaoundé Central Hospital, and non-obese controls from the general population between February and April 2011. Diabetic patients and pregnant women were excluded from the study. For all participants, data was collected on the sex and age. Height, waist and hip circumference to the nearest 0.5 cm were measured, as well as weight in light clothes to the nearest 0.1 kg. The body mass index (BMI) as weight in kg divided by height in meters squared (m^2^), and the waist-to-hip ratio as waist circumference (cm) divided by hip circumference (cm) were calculated. Obesity was defined as BMI ≥ 30 kg/m^2^. The resting blood pressures were measured using standardized procedures with an automatic sphygmomanometer Omron HEM-705 CP (Omron Corporation, Tokyo, Japan).

### Biochemical assays

Fasting plasma glucose (glucose oxidase–peroxidase method), serum cholesterol (cholesterol oxidase phenol-4-amino antipyrene peroxidase method), serum triglycerides (glycerol phosphatase oxidase phenol-4-amino antipyrene peroxidase method), and high-density lipoprotein (HDL)-cholesterol (cholesterol oxidase phenol-4-amino antipyrene peroxidase method) were measured on a spectrophotometer (UV Mini 1240) using Chronolab kits (Chronolab Systems, Barcelona, Spain). Low-density lipoprotein (LDL)-cholesterol was calculated using the Friedwald’s formula [14].

### DNA extraction and molecular genotyping

DNA was extracted from whole blood on filter paper by the Chelex method. Sixty-five participants (35 obese patients and 30 controls) were genotyped for *TCF7L2* rs12255372 by polymerase chain reaction–restriction fragment length polymorphism (PCR–RFLP). The *TCF7L2* rs12255372 (*G/T*) polymorphism was genotyped using the following primers: forward 5′-CTG GAA ACT AAG GCG TGA GG-3′, reverse 5′-GGG TCG ATG TTG TTG AGC TT-3′ (SIGMA-ALDRICH, St. Louis, Missouri, USA). A final reaction volume of 15 μL for the PCR was constituted which contained 100 ng of genomic DNA, 5 pmol of each primer, PCR buffer with 1 mmol/L of MgCl_2_, 100 μmol/L of each deoxynucleotide triphosphate (dNTP), 0.5 U of Hot Star Taq DNA polymerase (QIAGEN) and 7.8 µl of nuclease-free water. The PCR was carried out in a BIOMETRA T3 Thermal Cycler under the following conditions: 95 °C for 15 min, followed by 34 cycles of 95 °C for 30 s, 54 °C for 30 s, 72 °C for 30 s, and a final extension of 72 °C for 9 min. The PCR amplicons (346 bp) were then digested with *Thermus species* (*Tsp 5091*) restriction enzyme at 37 °C overnight. The reaction volume was set to 15 μl, containing 7 μl of amplicons, 1× NEB buffer1. The resulting products were separated by electrophoresis on a 3 % agarose gel using a 100 bp DNA ladder and visualized under a UV transilluminator.

### Statistical analyses

Allele and genotype frequencies in patients and control subjects were estimated by direct counting. Qualitative variables were analyzed by the Chi Square (χ^2^) test with Yates’ Continuity correction or the Exact test when appropriate using Epi Info version 6 (USD, Stone Mountain, USA). Quantitative variables were analysed by Mann–Whitney U test statistics using IBM SPSS for Windows version 20.0 (SPSS, Inc., Chicago, IL, USA). P < 0.05 was considered statistically significant and in case of multiple comparisons, the conservative Bonferroni correction was applied. The relative risks (RR) were calculated as odd ratios (OR) with 95 % confidence interval (CI) using Woolf’s formula.

### Ethical considerations

The study was granted approval by the National Ethical Review Board of the Cameroon Ministry of Public Health. Written informed consent was obtained from all the participants. The study was conducted in accordance with the Helsinki Declaration.

## Results

### Characteristics of the study population

**Fig. 1:**
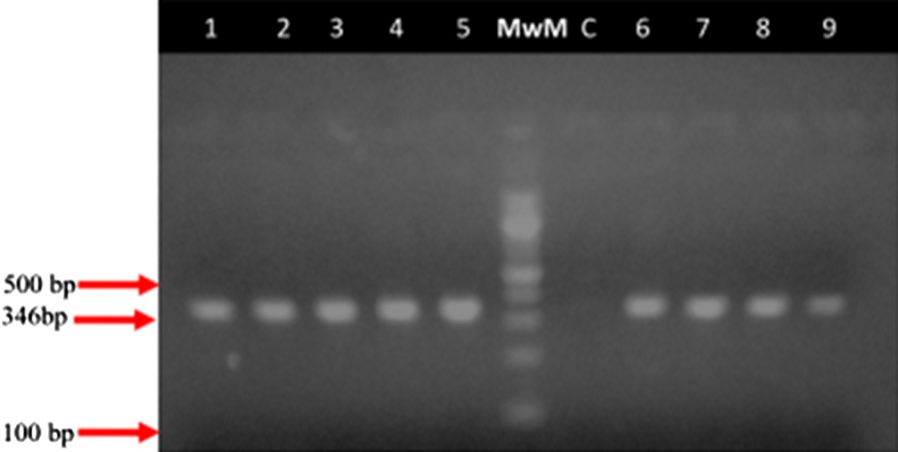
Electrophoregram of amplified rs 12255372 portion of the TCF7L2 gene. *C* negative control, *MwM* molecular weight marker (100 bp ladder), *1–9* tested samples, *bp* base pair

**Fig. 2:**
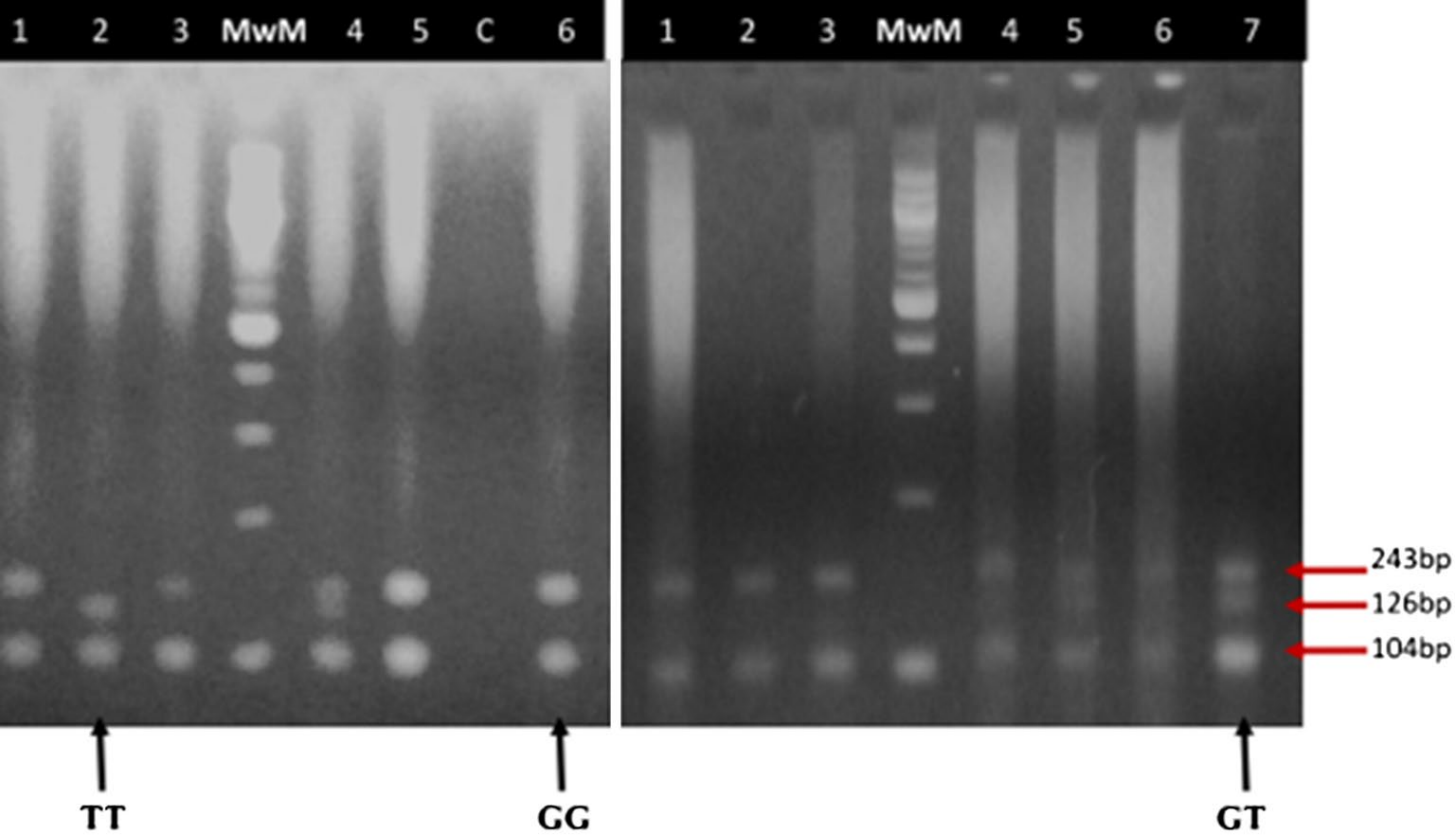
Electrophoregram of digested amplicons with Tsp 5091. *C* negative control, *MwM* molecular weight marker (100 bp ladder), *1–6* and *1–7* tested samples, *bp* base pair

Table 1 shows anthropometric features, blood pressure and metabolic profile of the study population. Significant differences between obese patients and health controls were observed for age (median age—46.00 vs 25.00 years, p < 0.0001), waist-to-hip ratio (median value—0.8600 vs 0.8100, p < 0.0001), diastolic blood pressure (median value—80.00 vs 68.00 mmHg, p < 0.0040) and BMI (median value—32.76 vs 22.39 kg/m^2^, p < 0.0001). All participants were positively genotyped. The respective fragments for the different genotypes were as follows. The wild type homozygote GG genotype had two band appearing at 143 and 104 bp; the mutant homozygote TT had two bands appearing at 126 and 104 bp and the mutant heterozygote GT had three bands appearing at 143, 126 and 104 bp. Fragments smaller than 100 bp migrated out of the gel and were not visualized (Figs. 1, 2).

**Table 1:** Characteristics of the study population

Characteristics	Non-obese (n = 30)	Obese (n = 35)	*P* value
Demographic			
Sex, % of male	33.3 %	8.6 %	0.028
Age (years)	25.00 (23.00–36.50)	46.00 (39.00–53.00)	<0.0001
Clinical			
WHR ratio	0.81 (0.77–0.84)	0.86 (0.83–0.93)	<0.0001
SBP (mmHg)	116.00 (106.00–125.00)	125.00 (117.00–141.00)	0.27
DBP (mmHg)	68.00 (61.75–85.00)	80.00 (75.00–88.00)	0.004
BMI (kg/m^2^)	22.39 (21.11–23.93)	32.76 (30.94–35.72)	<0.0001
Biological			
FPG (g/L)	90.00 (83.00–103.25)	96.00 (86.00–103.00)	0.392
TC (mg/dL)	171.45 (161.46–192.83)	181.94 (172.26–208.31)	0.093
HDL-C (mg/dL)	46.79 (42.71–50.16)	47.96 (43.70–51.72)	0.247
LDL-C (mg/dL)	100.05 (92.87–120.24)	106.97 (94.08–135.88)	0.490
TG (mg/dL)	125.29 (120.50–135.16)	131.80 (120.69–144.83)	0.193

Data are medians (interquartile range) unless otherwise stated

*WHR* waist-to-hip ratio, *SBP* systolic blood pressure, *DBP* diastolic blood pressure, *BMI* body mass index, *FPG* fasting plasma glucose, *TC* total cholesterol, *HDL* high density lipoprotein cholesterol, *LDL* low density lipoprotein cholesterol, *TG* triglycerides

### Genetic association assessment

The *TCF7L2* rs12255372 (G/T) SNP was in Hardy–Weinberg equilibrium in the general population (χ^2^ = 0.0500, p = 0.8300). The association between *TCF7L2* and obesity in Cameroon was determined by comparing the allele and genotype frequencies of rs12255372 (*G/T*) in the 35 obese patients and 30 healthy controls (Table 2). The allelic frequencies obtained in the general population were 18.35 % for the T allele and 81.65 % for the G allele. In the case–control analysis, no significant difference was observed in the allelic and genotypic frequencies between the obese and non-obese groups (χ^2^ = 0.0684, *p* = 0.7900 for allele and χ^2^ = 0.3720, *p* = 0.5400 for genotype).

**Table 2:** Case–control association analysis of *TCF7L2* rs12255372 (G/T) with obesity

*TCF7L2* rs12255372 G/T	Non-obese^a^	Obese^a^	Test statistics	*P* value	OR (95 % CI)
Alleles					
G	50 (83.3)	56 (80)	χ^2^ = 0.0684	0.79	0.51 (1.25–3.06)
T	10 (16.7)	14 (20)	χ^2^ = 0.0684	0.79	0.33 (0.80–1.96)
Total (2n)	60	70			
Genotypes					
G/G	21 (70)	22 (62.9)	χ^2^ = 0.118	0.73	1.38 (0.49–3.90)
G/T	8 (26.7)	12 (34.3)	χ^2^ = 0.155	0.69	0.70 (0.23–2.03)
T/T	1 (3.3)	1 (2.9)	χ^2^ = 0.372	0.54	1.17 (0.07–19.59)
Total (n)	30	35			

RR (relative risks) were calculated as odd ratios (OR) with 95 % CI using Woolf’s formula. When one of the entries was zero, OR was calculated using Woolf’s formula with Haldane’s modification

^a^Data are given as n (%) unless otherwise stated

### Genotype–phenotype correlation

Genotype–phenotype correlations were performed to investigate the association of *TCF7L2* rs12255372 and metabolic parameters in lean and obese individuals. These case–control analyses were performed according to demographic (sex and age at onset), clinical (body mass index, waist-to-hip ratio, systolic and diastolic blood pressure) and biological (fasting plasma glucose, total cholesterol, HDL, LDL and triacylglycerol) characteristics. T allele carriers in the obese group had a significantly higher triglyceride levels when compared to their non-obese counterparts (p < 0.0120).

## Discussion

*TCF7L2* has been associated with fasting plasma insulin, with glucose levels in type 2 diabetes patients to some extent with BMI [10, 15, 16], and with adipogenesis [9]. It is one of the genes that have been identified as possible determinants of diabetes. Data on genetic susceptibility of African populations to chronic disease are very scarce [17]. The aim of this study was to assess the possible association of the *TCF7L2* rs12255372 polymorphism with obesity and weight-related traits in a Cameroonian population. This case–control association analysis demonstrates the absence of association between the polymorphism and obesity. This finding is similar to those of Cauchi et al. [10] and Saxena et al. [18] who also found no genetic association between *TCF7L2* and obesity in European and US populations, respectively, especially for the rs7903146 and rs10885406 polymorphisms. However, some other studies reported a tendency for negative association with BMI [10, 19]. Nevertheless, these results are different from those of Klunder et al. that showed a protective association between rs12255372 polymorphism and obesity in Mexican children, while a deleterious effect of this variant was observed in diabetic adults [11]. Moreover, the background frequencies of the *TCF7L2* rs12255372 alleles and genotypes were different between the Cameroonian and Mexican populations indicating genetic heterogeneity across populations and ancestries on this locus. The frequency of this risk allele has been observed to be highest in a study in Caucasians (77 %) with 42 % in cases (diabetics) and 35 % in controls [20] and lowest in Asians (1 %) with 0.6 % in cases and 0.4 % in controls [21].

Studies have shown that some variants of *TCF7L2* are associated with impaired lipid metabolism, especially a trend towards higher triglycerides, with an adverse effect on cardiovascular risk and stroke incidence [22, 23]. Meta-analyses have been conducted to confirm the association from individual studies. These studies (meta-analyses) further support the association between TCF7L2 gene and metabolic diseases including diabetes and obesity. Among the common studied allele, rs12255372 variant of the TCF7L2 gene is significantly associated with susceptibility to T2DM in the global population [24] while the other allele (rs7903146) is associated with metabolic syndrome [25] of which dyslipidemia is a predictor. *TCF7L2* rs12255372 has also been found to be associated with high serum triglycerides and was differentially expressed in adipose tissue in families with familial combined hyperlipidaemia [26]. Genotype–phenotype correlation assessment in our study revealed that the gene was preferentially associated with high triglyceride levels. Carriers of the T risk allele had a significantly higher triglyceride level when compared to carriers of the G allele (p < 0.0120). This finding suggests a possible association between the *TCF7L2* rs12255372 variant and cardiovascular risk in the Cameroonian population. Further studies are therefore required to confirm these findings and then investigate the nature of the association.

## Conclusion

*TCF7L2* rs12255372 was not associated with obesity in this Cameroonian population, but was associated with higher triglyceride levels in obese patients. Due to the small sample size of the study and the lack of power to detect an association, other studies carried with a larger sample size and matched for age and sex are required to confirm these findings.
